# Contributing Factors to Safety: What Hospitalized Patients Can Tell Us? A Cross‐Sectional Study

**DOI:** 10.1002/hpm.3945

**Published:** 2025-05-16

**Authors:** Franciely Daiana Engel, Caroline Cechinel‐Peiter, Diovane Ghignatti da Costa, José Luis Guedes dos Santos, Alacoque Lorenzini Erdmann, Elena Bohomol, Chantal Backman, Ana Lúcia Schaefer Ferreira de Mello

**Affiliations:** ^1^ Graduate Program in Nursing Health Sciences Center Federal University of Santa Catarina Florianópolis Brazil; ^2^ School of Nursing Faculty of Health Sciences University of Ottawa Ottawa Canada; ^3^ Graduate Program in Nursing Nursing School Federal University of São Paulo São Paulo Brazil

**Keywords:** hospital, patient outcome assessment, patient participation, patient safety

## Abstract

**Background:**

Brazil has the second‐highest COVID‐19 mortality rate worldwide. While there are currently no guidelines for involving patients in their own safety, recognising patients' valuable feedback can be decisive for the safety and quality of healthcare. Thus, this study aimed to describe the patient feedback on factors contributing to safety in patients hospitalised with COVID‐19 in Brazil and to examine associations with patient sociodemographic and clinical characteristics.

**Methods:**

A cross‐sectional study was conducted in nine Brazilian university hospitals. Data collection using the Patient Measure of Safety (PMOS) questionnaire was conducted by telephone with 447 patients who recovered from COVID‐19. Descriptive and multilevel linear regression models were used to verify the sociodemographic characteristics associated with PMOS.

**Results:**

Patients felt safer when they accessed healthcare resources, when health professionals communicated well, and when they had good teamwork skills. Sociodemographic and clinical factors influenced the patient's perception of safety. A lower perception of safety was observed among patients aged 18–39 years old, of mixed race, and who had more than six symptoms during hospitalisation. Higher perceptions of safety were identified among patients with higher education, who lived in the countryside, and who required admission to the ICU.

**Conclusions:**

This study highlighted the potential for patients to become crucial allies in ensuring safety within hospital settings by providing insights into their care, and how sociodemographic characteristics can influence the perception of safety.

**Summary**

- Communication, teamwork, and equipment were favourable for patient safety.
- Young, mixed‐race, and highly symptomatic patients perceived lower safety.
- ICU admission and higher education positively influenced safety perceptions.
- Patients' feedback highlights gaps in safety and informs quality improvement.

## 1 Introduction

Patient involvement in safety contributes to creating a safe environment beyond monitoring and data production. Patients can provide valuable information about safety during their health care episodes, including their concerns with physical comfort (e.g., noise, lighting), their fears or uncertainties about their care plan, and their experiences with procedure or treatment delays, which can have a direct impact on their quality of care [1]. Thus, it is relevant to incorporate patients' perspectives in safety assessments, as they can reveal gaps, highlight opportunities for improvement [2], and influence their own safety outcomes [3].

When patients and their families are actively involved in care, they significantly contribute to incident prevention by remaining vigilant about healthcare risks. Collaboration between patients and healthcare professionals improves the quality of care and safety in the hospital environment, strengthens mutual trust, and enhances health outcomes and experiences for the patient [4]. Also, positive healthcare experiences can make patients more attentive and committed to ensuring their safety during hospitalisation [5].

The COVID‐19 pandemic has brought additional stressors to the healthcare system, such as resource scarcity and increased workloads. These stressors have decreased patient satisfaction [6, 7], and increased concerns about patient safety and quality of care [8]. Particularly, Brazil has reported over 37 million confirmed COVID‐19 cases and more than 704,000 COVID‐19 deaths, with a case fatality rate of 1.9% and a mortality rate of 335.6 per 100,000 inhabitants [9, 10]. The country has a universal public healthcare system, which faces challenges related to socioeconomic disparities, resource scarcity, and integration of services, along with various barriers to healthcare access [11]. Although Brazil has included patient involvement initiatives within its health policies, these practices remain in an early stage [12]. Healthcare professionals still face challenges such as limited training, shortages of material and human resources, and gaps in health literacy [13].

Considering the challenges that the pandemic has brought to health services, the context of Brazil's health crisis, and the importance of patient involvement in their care and safety, the objectives of our study were to describe patient feedback on factors contributing to safety in patients hospitalised with COVID‐19 in Brazil and to examine associations with patient sociodemographic and clinical characteristics.

## 2 Method

### 2.1 Study Design

This was a cross‐sectional study conducted between April to December 2021. It was part of the multicenter project “Evaluation of nursing care for patients with COVID‐19 in Brazilian university hospitals”. This research was approved by the Human Research Ethics Committee (protocol number: 38,912,820.3.1001.0121). The STROBE checklist for cross‐sectional studies was used to report this study [14].

### 2.2 Study Setting and Population

The study was conducted in nine Brazilian university hospitals across all five regions (North, Northeast, South, Southeast, and Midwest), chosen for their status as COVID‐19 reference centres and existing research partnerships. The study population consisted of adult patients hospitalised with COVID‐19 and subsequently discharged home.

### 2.3 Sampling

The sample size was calculated based on the absolute margin of error to estimate the mean values. We used the Winpepi (PEPI‐for‐Windows) programme, version 11.65, with a confidence level of 95%, a margin of error of 0.3 points, and a standard deviation of 1.28, as reported by Taylor et al. [15]. This procedure resulted in a required sample size of 668 participants.

### 2.4 Selection Criteria

Inclusion criteria consisted of patients diagnosed with COVID‐19, aged 18 years or older, fluent in Brazilian Portuguese, hospitalised for at least 72 h, discharged home, and with a minimum of seven days post‐discharge. Patients who were severely debilitated or had been readmitted to the hospital at the time of the phone survey were excluded from the study.

### 2.5 Data Collection

Data collection was conducted between April and December 2021. The participating hospitals provided contact information of patients hospitalised due to COVID‐19 complications and were discharged home. Phone calls were made to these patients at least 7 days after discharge. Patients were introduced to the study, provided informed consent, and then were interviewed. The team of interviewers included nursing professors, healthcare professionals, undergraduate, and graduate students who received appropriate training for conducting the survey. For more information, see the Data Collection article [16].

A questionnaire was used to collect information on participants' sociodemographic characteristics and clinical signs and symptoms presented during hospitalisation. The Patient Measure of Safety (PMOS) questionnaire was used to identify factors contributing to patient safety [17]. All phone calls were audio recorded and randomly audited to verify patient responses with the recorded questionnaires.

### 2.6 Study Variables

#### 2.6.1 Dependent Variable

The factors contributing to patient safety were measured by the PMOS questionnaire. The PMOS was structured following The Yorkshire Contributory Factors Framework [18], developed in England in 2011 (Cronbach's α (range 0.66–0.89)) [18] and validated for use in Brazil in 2018 (Cronbach's α (range 0.55–0.87)) [17]. The PMOS consists of 44 questions, organised into nine domains: (1) Communication and teamwork (9 items); (2) Care organization and planning (5 items); (3) Access to resources (4 items); (4) Ward type and presentation (12 items); (5) Information flow (3 items); (6) Team assignments and responsibilities (4 items); (7) Team training (2 items); (8) Equipment (2 items); and (9) Delays (2 items). Responses are structured on a Likert scale, ranging from 1 (totally disagree) to 5 (totally agree), with the options “not applicable” and “prefer not to answer” [17]. Domain‐specific scores were calculated based on the average of their respective items. Domain scores were computed using the criterion that participants responded to at least two items within the domain. Overall scores were calculated based on the average of the domains. A total of 10 scores were generated, ranging from 1 to 5, with higher scores indicating greater perceptions of patient safety.

#### 2.6.2 Independent Variables

The categorical variables were Sex (Female; Male); Education level (No education; Completed primary school; Completed high school; Completed higher education); Race (Caucasian; Black; Mixed race; Other); Household income (No income; Up to 2 monthly minimum wages (MMW); > 2MMW to ≤ 5MMW; > 5MMW to ≤ 10MMW; More than 10MMW); In 2020, the MMW in Brazil was R$1.045,00 which was equivalent to approx. US$190.00; Location (City Capital; Countryside); ICU Hospitalisation (Did not require admission to the ICU; Required admission to the ICU); Use of mechanical ventilation (Yes; No); Smoking history (Non‐smoker; Smoker; Ex‐smoker); Pre‐existing comorbidities (No comorbidities; One or two comorbidities; Three or more comorbidities, considering: chronic respiratory disease; systemic arterial hypertension; cardiovascular diseases; diabetes mellitus; kidney diseases; obesity; cancer); and Signs and symptoms presented during hospitalisation (Up to three symptoms; Four to five symptoms; Six to seven symptoms; Eight or more symptoms, considering: fever; fatigue; shortness of breath; cough; loss of smell and taste; headache; body pain; nausea and vomiting; diarrhoea). The continuous variables included were Age (in years), and Length of hospital stay (in complete days).

### 2.7 Data Analysis

Descriptive statistics included central tendency (mean) and dispersion (standard deviation) for continuous variables, and absolute/relative frequencies for categorical variables. Multilevel linear regression models assessed sociodemographic factors associated with PMOS scores, accounting for clustering (patients nested within hospitals). Independent variables, chosen based on theoretical predictors of patient safety, were entered simultaneously for mutual adjustment. All models included a random hospital intercept and were tested for multilevel assumptions. Asymmetric residuals in some models prompted the use of bootstrap techniques (2000 resamples by hospital clusters) to correct standard errors and 95% confidence intervals. Analyses were performed with STATA 14.2. A statistical significance of 5% (*p* < 0.05) was considered in all analyses.

## 3 RESULTS

Among the 3950 patient contacts received from university hospitals, 3106 met the eligibility criteria based on hospital‐registered information. Common reasons for ineligibility were hospital stays lasting less than 72 h (*n* = 270) and post‐discharge patient mortality (*n* = 202). Additionally, 2567 were lost to follow‐up, primarily due to unsuccessful contacts after the collection period ended (*n* = 1867) or discrepancies in phone numbers (*n* = 403). A total of 262 patients declined to participate in the study. Overall, 539 patients participated in the study, representing 80.6% of the estimated sample size, and 447 had complete data.

### 3.1 Sociodemographic Characteristics

The participants' mean age was 51.7 (SD 15.3) years, with a predominance of adults aged 40–59 years; 51.5% (*n* = 230) were female, and 46.8% (*n* = 209) identified as mixed race. Regarding education, 44.3% (*n* = 198) had completed high school, and 40.7% (*n* = 182) had a monthly family income of up to two minimum wages. Most participants were non‐smokers (64.9%; *n* = 290) and had one or two comorbidities (51.5%; *n* = 230). The most prevalent comorbidities were hypertension (46.5%; *n* = 208) and diabetes mellitus (29.3%; *n* = 131).

### 3.2 Clinical Characteristics

A total of 32.2% (*n* = 144) presented six to seven symptoms during the hospitalisation. The two most frequent symptoms were dyspnea (78.3%; *n* = 350) and fatigue (75.1%; *n* = 336). According to the interquartile range, half of the participants were hospitalised for 8–25 days 46.3% (*n* = 207) of patients were admitted to the ICU, and 23.7% (*n* = 106) required mechanical ventilation. See Table 1 for more details.

**TABLE 1:** Sociodemographic characteristics.

Variables	Overall
	*N* = 447
Sex n(%)	
Male	217(48.5)
Female	230(51.5)
Age, mean(SD)	51.7(15.3)
Age rate n(%)	
18–39 years old	101(22.6)
40–59 years old	201(45.0)
≥ 60 years old	145(32.4)
Ethnicity n(%)	
Caucasian	174(38.9)
Black	54(12.1)
Mixed race	209(46.8)
Other	10(2.2)
Educational levels n(%)	
Incomplete elementary school	107(23.9)
Elementary school	80(17.9)
High school	198(44.3)
Higher education	62(13.9)
Household income n(%)	
No income	17(3.8)
Up to 2 MMW^†^	182(40.7)
> 2MMW to ≤ 5MMW	146(32.7)
> 5MMW to ≤ 10MMW	43(9.6)
More than 10 MMW	13(2.9)
I prefer not to respond to this question	46(10.3)
Days of hospitalisation, median(P25‐P75)^‡^	13.0 (8.0–25.0)
ICU admission n(%)	
Did not require admission to the ICU	240(53.7)
Required admission to the ICU	207(46.3)
Use of mechanical ventilation n(%)	
No mechanical ventilation needed	341(76.3)
Required mechanical ventilation	106(23.7)
Smoking history n(%)	
Non‐smoker	290(64.9)
Smoker	16(3.6)
Former smoker	141(31.5)
History of comorbidities n(%)	
No comorbidities	101(22.6)
One or two comorbidities	230(51.5)
Three or more comorbidities	116(26.0)
Symptoms during hospitalisation n(%)	
Up to three symptoms	101(22.6)
Four to five symptoms	116(26.0)
Six to seven symptoms	144(32.2)
Eight or more symptoms	86(19.2)
Location of the city	
City capital	285(63.8)
Countryside	162(36.2)

^†^ In the year 2020, the monthly minimum wage (MMW) in Brazil was R$1.045,00 which was equivalent to approx. US$190,00.

^‡^ P25‐P75 ‐ lower and upper quartile.

**FIGURE 1:**
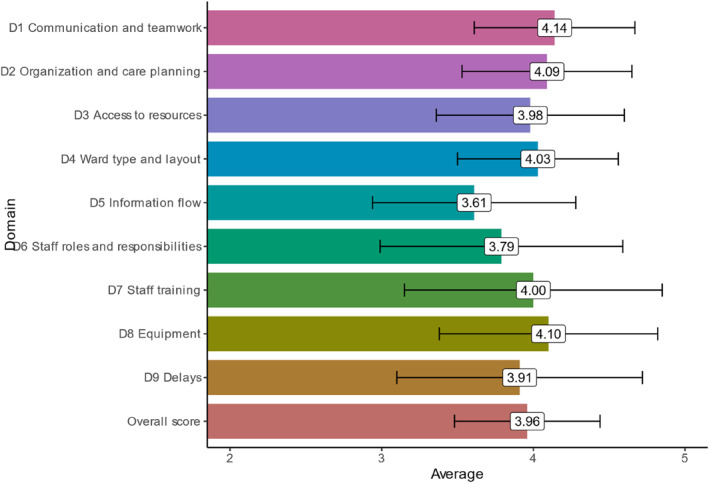
PMOS score according to PMOS domains.

The results of the PMOS scores showed that the most favourable factors for patient safety were D1 “Communication and teamwork”, with a mean score of 4.14 (SD 0.53), and D8 “Equipment”, with a mean score of 4.10 (SD 0.72). However, the factors that needed attention and were perceived as less favourable for patient safety were D5 “Information flow”, with a mean score of 3.61(SD 0.67), and D6 “Staff roles and responsibilities”, with a mean score of 3.79 (SD 0.80). The PMOS Scores can be found in Figure 1.

The adjusted models showed a significant association between sociodemographic/clinical characteristics and PMOS scores (Table 2). *Age:* It was identified that young adults aged 18 to 39 had a lower mean score (−0.24 [−0.37, −0.11]) compared to adults aged 40 to 59 in the overall score and domains D1 “Communication and teamwork” (0.24 [−0.39, −0.10]), D2 “Organization and care planning” (−0.18 [−0.34, −0.02]), D3 “Access to resources” (−0.33 [−0.51, −0.15]), D5 “Information flow” (−0.27 [−0.44, −0.10]), D6 “Staff roles and responsibilities” (−0.22 [−0.42, −0.02]), D8 “Equipment” (−0.34 [−0.55, −0.13]) and D9 “Delays” (−0.27 [−0.49, −0.05]).

**TABLE 2:** Sociodemographic data according to PMOS dimensions.

Variables (*n* = 447)	Overall	D1	D2	D3	D4
	β (95%IC)	β (95%IC)	β (95%IC)	β (95%IC)	β (95%IC)
Sex					
Male	Ref.	Ref.	Ref.	Ref.	Ref.
Female	0.01 [−0.07,0.09]	−0.00 [−0.09,0.09]	−0.02 [−0.12,0.07]	0.03 [−0.09,0.14]	0.00 [−0.09,0.09]
Age rate					
18–39 years old	**−0.24 [−0.37,−0.11]**	**−0.24 [−0.39,−0.10]**	**−0.18 [−0.34,−0.02]**	**−0.33 [−0.51,−0.15]**	−0.11 [−0.23,0.02]
40–59 years old	Ref.	Ref.	Ref.	Ref.	Ref.
≥ 60 years old	−0.04 [−0.13,0.06]	−0.01 [−0.12,0.09]	0.02 [−0.09,0.13]	−0.07 [−0.19,0.06]	0.03 [−0.08,0.13]
Ethnicity					
Caucasian	Ref.	Ref.	Ref.	Ref.	Ref.
Black	−0.01 [−0.16,0.14]	−0.02 [−0.19,0.14]	0.07 [−0.10,0.24]	−0.01 [−0.21,0.19]	0.02 [−0.14,0.18]
Mixed race	−0.07 [−0.16,0.03]	−0.07 [−0.18,0.04]	−0.07 [−0.19,0.05]	−0.03 [−0.15,0.09]	−0.00 [−0.10,0.10]
Other	−0.10 [−0.47,0.27]	−0.09 [−0.48,0.29]	−0.27 [−0.78,0.24]	−0.21 [−0.69,0.28]	−0.00 [−0.39,0.38]
Educational levels					
Incomplete elementary school	Ref.	Ref.	Ref.	Ref.	Ref.
Elementary school	0.11 [−0.03,0.25]	0.12 [−0.03,0.26]	0.06 [−0.10,0.22]	0.14 [−0.03,0.31]	**0.23 [0.08,0.37]**
High school	0.11 [−0.01,0.22]	0.12 [−0.00,0.24]	0.08 [−0.04,0.21]	0.03 [−0.11,0.17]	0.11 [−0.02,0.24]
Higher education	0.05 [−0.11,0.21]	0.03 [−0.15,0.21]	0.04 [−0.15,0.23]	−0.02 [−0.23,0.19]	0.09 [−0.08,0.25]
Weeks of hospitalisation	−0.01 [−0.03,0.01]	0.01 [−0.02,0.03]	−0.00 [−0.03,0.02]	−0.02 [−0.05,0.01]	−0.01 [−0.03,0.01]
ICU admission					
Did not require admission to the ICU	Ref.	Ref.	Ref.	Ref.	Ref.
Required admission to the ICU	0.08 [−0.03,0.20]	0.09 [−0.03,0.22]	0.07 [−0.07,0.21]	0.04 [−0.11,0.18]	**0.11 [0.01,0.22]**
Use of mechanical ventilation					
No mechanical ventilation needed	Ref.	Ref.	Ref.	Ref.	Ref.
Required mechanical ventilation	−0.08 [−0.21,0.04]	−0.10 [−0.24,0.03]	−0.11 [−0.27,0.04]	−0.07 [−0.24,0.10]	−0.14 [−0.28,0.01]
History of comorbidities	−0.01 [−0.04,0.03]	−0.03 [−0.07,0.01]	−0.00 [−0.04,0.04]	−0.02 [−0.07,0.02]	−0.02 [−0.06,0.02]
Symptoms during hospitalisation					
Up to three symptoms	Ref.	Ref.	Ref.	Ref.	Ref.
Four to five symptoms	0.06 [−0.06,0.18]	0.10 [−0.03,0.24]	0.09 [−0.06,0.23]	0.10 [−0.05,0.26]	0.03 [−0.11,0.17]
Six to seven symptoms	0.00 [−0.12,0.12]	0.05 [−0.09,0.19]	0.02 [−0.13,0.17]	−0.01 [−0.17,0.14]	0.01 [−0.11,0.14]
Eight or more symptoms	−0.10 [−0.24,0.03]	−0.04 [−0.19,0.12]	−0.09 [−0.27,0.08]	−0.05 [−0.22,0.12]	−0.11 [−0.25,0.04]
Location of the city					
City capital	Ref.	Ref.	Ref.	Ref.	Ref.
Countryside	0.09 [−0.00,0.19]	**0.13 [0.02,0.24]**	0.11 [−0.01,0.23]	0.08 [−0.06,0.21]	0.07 [−0.04,0.19]

**Variables (n = 447)**	**D5**	**D6**	**D7**	**D8**	**D9**
	**β (95%IC)**	**β (95%IC)**	**β (95%IC)**	**β (95%IC)**	**β (95%IC)**
Sex					
Male	Ref.	Ref.	Ref.	Ref.	Ref.
Female	0.00 [−0.13,0.13]	−0.07 [−0.21,0.07]	0.10 [−0.05,0.25]	0.00 [−0.13,0.13]	0.03 [−0.12,0.18]
Age rate					
18–39 years old	**−0.27 [−0.44,‐0.10]**	**−0.22 [−0.42,‐0.02]**	−0.17 [−0.40,0.07]	**−0.34 [−0.55,‐0.13]**	**−0.27 [−0.49,‐0.05]**
40–59 years old	Ref.	Ref.	Ref.	Ref.	Ref.
≥ 60 years old	−0.06 [−0.21,0.09]	−0.06 [−0.23,0.12]	0.01 [−0.15,0.18]	**−0.14 [−0.29,‐0.00]**	−0.05 [−0.23,0.12]
Ethnicity					
Caucasian	Ref.	Ref.	Ref.	Ref.	Ref.
Black	0.17 [−0.02,0.36]	0.06 [−0.21,0.33]	−0.05 [−0.31,0.21]	−0.20 [−0.45,0.05]	−0.13 [−0.40,0.14]
Mixed race	−0.07 [−0.20,0.07]	0.05 [−0.12,0.22]	**−0.20 [−0.37,‐0.03]**	**−0.17 [−0.30,‐0.03]**	−0.01 [−0.18,0.15]
Other	−0.08 [−0.55,0.39]	0.36 [−0.08,0.81]	−0.45 [−0.94,0.03]	−0.11 [−0.65,0.44]	−0.07 [−0.54,0.40]
Educational levels					
Incomplete elementary school	Ref.	Ref.	Ref.	Ref.	Ref.
Elementary school	0.03 [−0.17,0.23]	0.23 [−0.00,0.46]	0.04 [−0.22,0.30]	0.06 [−0.12,0.24]	0.10 [−0.14,0.35]
High school	0.09 [−0.07,0.25]	**0.32 [0.14,0.51]**	0.07 [−0.13,0.26]	−0.06 [−0.22,0.10]	0.17 [−0.02,0.36]
Higher education	−0.01 [−0.22,0.21]	**0.31 [0.08,0.54]**	−0.03 [−0.34,0.28]	−0.01 [−0.23,0.22]	−0.03 [−0.29,0.24]
Weeks of hospitalisation	−0.02 [−0.05,0.00]	**0.04 [0.01,0.07]**	−0.00 [−0.04,0.03]	−0.00 [−0.03,0.03]	**−0.04 [−0.06,‐0.01]**
Hospitalisation on ICU					
Did not require admission to the ICU	Ref.	Ref.	Ref.	Ref.	Ref.
Require admission to the ICU	**0.16 [0.00,0.31]**	−0.01 [−0.21,0.19]	0.05 [−0.15,0.24]	0.04 [−0.13,0.21]	**0.19 [0.00,0.38]**
Use of mechanical ventilation					
No mechanical ventilation needed	Ref.	Ref.	Ref.	Ref.	Ref.
Require mechanical ventilation	−0.04 [−0.22,0.13]	0.02 [−0.19,0.24]	−0.08 [−0.32,0.16]	−0.09 [−0.29,0.11]	−0.19 [−0.42,0.04]
History of comorbidities	−0.02 [−0.07,0.02]	0.04 [−0.02,0.10]	−0.00 [−0.06,0.06]	0.02 [−0.03,0.07]	−0.02 [−0.07,0.04]
Symptoms during hospitalisation					
Up to three symptoms	Ref.	Ref.	Ref.	Ref.	Ref.
Four to five symptoms	−0.01 [−0.19,0.16]	0.07 [−0.16,0.30]	−0.11 [−0.29,0.08]	**0.21 [0.05,0.37]**	0.04 [−0.16,0.24]
Six to seven symptoms	0.05 [−0.13,0.22]	0.16 [−0.05,0.36]	**−0.20 [−0.39,‐0.01]**	−0.03 [−0.19,0.13]	−0.05 [−0.26,0.17]
Eight or more symptoms	−0.14 [−0.32,0.05]	−0.03 [−0.28,0.22]	**−0.27 [−0.50,‐0.03]**	−0.15 [−0.36,0.06]	−0.05 [−0.29,0.18]
Location of the city					
City capital	Ref.	Ref.	Ref.	Ref.	Ref.
Countryside	−0.01 [−0.15,0.13]	**0.19 [0.02,0.35]**	0.14 [−0.03,0.32]	0.08 [−0.07,0.23]	0.09 [−0.08,0.27]

*Note:* Bold values are significant at *p* < 0.05; Ref., reference category. D1, Communication and teamwork; D2, Organization and care planning; D3, Access to resources; D4, Ward type, and layout; D5, Information flow; D6, Staff roles, and responsibilities; D7, Staff training; D7, Equipment; D9, Delays.

### 3.3 Ethnicity

Differences were also identified in ethnicity, where mixed‐race individuals rated 0.20 lower in mean score in domains D7 “Team training” (−0.20 [−0.37, −0.03]) and 0.17 lower in domain D8 ” Equipment (−0.17 [−0.30, −0.03]). *Education:* For domain D4 “Type and presentation of the ward”, patients who completed elementary school obtained an average PMOS score of 0.23 higher compared to those with incomplete elementary education (0.23 [0.08, 0.37]). For domain D6 “Staff roles and responsibilities”, patients with education above the elementary school had a higher score compared to patients with incomplete elementary education. *Place of residence:* Patients who lived in countryside areas had a higher average score of 0.13 in domain D1 “Communication and teamwork” (0.13 [0.02, 0.24]) and 0.19 in domain D6 “Staff roles and responsibilities” (0.19 [0.02, 0.35]).

### 3.4 Length of Hospital Stay

For each additional week of hospitalisation, the average PMOS score increased by 0.04 in domain D6 “Staff roles and responsibilities”(0.04 [0.01,0.07]). However, in domain D9 “Delays”, for every week of hospitalisation, the average PMOS score decreased by 0.04 (−0.04 [−0.06, −0.01]). *ICU hospitalisation:* Patients who were hospitalised in the ICU had a higher average score of 0.11 in domain D4 “Type and presentation of the ward” (0.11 [0.01, 0.22]), 0.16 in domain D5 “Information flow” (0.16 [0.00, 0.31]), and 0.19 in domain D9 “Delays” (0.19 [0.00, 0.38]) compared to those who did not require an ICU admission.

### 3.5 Presentation of Symptoms During Hospitalisation

Patients with six or more symptoms had a lower average score in domain D7 “Team training” (six to seven symptoms (−0.20 [−0.39, −0.01]) and eight or more symptoms (−0.27 [−0.50, −0.03])) compared to those with fewer than three symptoms. Also, patients who had between four and six symptoms had a higher average score of 0.21 in domain D8 “Equipment” (0.21 [0.05, 0.37]) compared to those who had fewer than three symptoms. No differences were found between sex, use of mechanical ventilation, history of comorbidities, and PMOS scores. See Table 2 for more details.

## 4 Discussion

In this study, we described the patients' feedback on factors contributing to safety during their hospitalisation for COVID‐19 in Brazil, and we examined associations with their sociodemographic and clinical characteristics. The favourable contributing factors to patient safety were related to communication, teamwork, and equipment aspects. This indicates that patients identified good communication as a key element that positively impacted their safety and the availability and use of healthcare equipment. This also aligns with a patient‐centred care approach, where clear and compassionate communication directly influences patient trust [19] and perceived safety [20]. However, less favourable contributing factors were the lack of information flow and difficulties in identifying staff roles and responsibilities. Moreover, even though the communication between health professionals was considered adequate, there were divergences between the information health professionals exchanged with patients and families. Patients were unsure who the healthcare professionals were responsible for their care.

By examining sociodemographic data, hospitalized COVID‐19 patients in Brazil were predominantly female, mixed race, with completed high school education, at least one comorbidity, and a family income of no more than two minimum wages. This aligns with findings in studies from other countries, further underscoring the role of health and socioeconomic disparities as pivotal factors influencing the risk of contracting COVID‐19 and requiring hospitalization [21, 22, 23].

As health inequities are presented in the country, differences were identified in the perception of the contributing factors to patient safety according to the patient's sociodemographic characteristics. Age and socioeconomic status impact the perception of healthcare and quality of care [24], which is in line with the results of our study. Age stood out in almost all domains investigated, resulting in a lower perception of patient safety in young adult patients. Furthermore, mixed‐race patients gave lower scores on “staff training” and “equipment”. In this sense, it is noteworthy that ethnic minorities are considered at greater risk for adverse events [25, 26], so capturing their feedback can be relevant to implementing more oriented preventive measures.

In Brazil, most general high‐capacity hospitals are in the capital cities and metropolitan regions. During the COVID‐19 pandemic, there was a need to transfer critically ill patients from rural and remote areas to urban centers due to the availability of ICU beds [27]. At the national level, priorities were listed in health contingency plans, including inter‐hospital transport regulation and the health system coordination following the complexity and the localization of health services provision [28]. In our study, those who lived in the countryside had better perceptions of “communication and teamwork” and “staff roles and responsibilities.” The same was perceived by patients with higher education levels and more extended hospital stays. The staff attitudes may have influenced the safety perceptions of patients who needed intensive care, considering that patients who were hospitalized in the ICU rated domains “ward type and layout,” “information flow,” and “delays” more positively. Studies that identified levels of safety culture in Brazilian hospitals during the pandemic showed that the highest‐scored domains were teamwork and supervisors' actions to promote patient safety [29].

It is recognized that nurses' positive attitudes toward patient safety have the potential to reduce the incidence of adverse events and improve patient outcomes [30]. Moreover, patient involvement in their safety constitutes strategies within nursing practice operating across different levels of care. Nurses can involve patients at the individual level, engaging them in the decision‐making process and establishing care objectives, as well as engage the patients at the organizational level aiming to enhance the quality of care provided within the institution. Additionally, patients and their families can contribute to formulating organizational actions and institutional policies, helping to improve healthcare quality [31].

This study showed that the longer the patient stayed in the hospital, the greater the patient's perception of delays in care, as indicated by the domain 9 scores. However, this could have been due to both organizational factors within the institution and the time the patient had to perceive this impact on healthcare. Additionally, individual patient factors may have also influenced their perception of safety, such as patients who experienced more than six symptoms during their hospitalization, having lower scores in the domains of “staff training” and “delays”. This aligns with the literature that highlights individual factors, such as the presence of comorbidities and severity of the health condition, with the occurrence of patient safety events [32, 33].

In this study, we observed that sociodemographic factors influenced the perception of safety during COVID‐19 hospitalization, and the institutional vulnerabilities were predominantly identified in “information flow” and “staff roles and responsibilities”. Institutions exhibiting weaknesses in patient communication demonstrated poorer patient engagement indicators in their care. Empowering patients to assume a more active role in decision‐making regarding their care holds potential for improved health outcomes [31]. Furthermore, it is crucial to highlight that patient safety encompasses emotional aspects, which leads to a broader discussion between the patient's safety per se and the patient's feeling safe while receiving healthcare [34].

Our findings highlight the importance of addressing gaps in information flow and clarifying staff roles and responsibilities to improve patient safety. To improve the flow of information, tools such as ISBAR (Introduction, Situation, Background, Assessment, Recommendation) have been shown to improve patient safety and quality of care [35]. In addition, patient‐centered education initiatives can ensure patients receive clear, consistent, and timely information about their care [36]. Clearly defining team roles and informing patients about the composition and functions of the multidisciplinary team can minimize patients' uncertainty about who is responsible for their care and promote a scenario in which patients can participate in the decision‐making [37].

In this study, patients identified the factors that contribute to patient safety, enabling the delineation of key elements that require attention. This underscores that patient input can enhance the analysis of quality of care and adherence to safety protocols, even in the context of healthcare crises. In addition to serving as a source of information, patient involvement in their care can significantly contribute to their safety [4, 38].

Brazil is the country with the second highest number of deaths due to COVID‐19 [10], and it is clear that the population most affected was due to social vulnerability, above issues such as age and chronic diseases [39]. This study has shown how sociodemographic aspects are associated with the perception of factors contributing to patient safety. Therefore, equity‐focused strategies can promote patient safety by addressing different realities and social determinants. Including experts in racism, intersectionality, and systemic oppression on the multi‐professional team, as well as patient safety equity measures, can enhance equity‐focused patient safety efforts [40].

This multicenter study involved a partnership with nine Brazilian federal universities. Thus, it was possible to investigate patient safety from the patients' perspectives nationally for the first time in Brazil, highlighting its relevance in representing developing countries. The statistical approach was robust and aligned with the study design. Multilevel analyses were performed to account for the hierarchical structure of data in which patients were nested within hospitals. Such an approach accounts for the heterogeneity of hospitals and provides more reliable estimates when compared to traditional linear regression (i.e., Ordinary Least Squares).

While national representation was not achieved, sample heterogeneity was ensured by representing different regions of the country. It was challenging to collect data by telephone, considering people's fear of answering calls and answering surveys, but we used several strategies to minimize this impact. Information about the data collection process is available for consultation [16].

## 5 Conclusion

Our study revealed the factors contributing to the safety of COVID‐19 patients hospitalised with COVID‐19 in Brazil based on their perceptions. Patients felt safer when they accessed healthcare resources, when health professionals communicated well, and when they had good teamwork skills. Sociodemographic and clinical factors influenced the patient's perception of safety. A lower perception of safety was observed among patients aged 18–39 years old, of mixed race, and who had more than six symptoms during hospitalisation. Higher perceptions of safety were identified among patients with higher education who lived in the countryside and required admission to the ICU.

This study highlighted the potential for patients to become crucial allies in ensuring safety within hospital settings by providing insights into their care. Also, the results claim attention to the need to enhance communication between healthcare professionals and patients to improve safety. Patient safety is more than ensuring materials and equipment for treatment and care. Considering the subjectivities presented in this study, such as communication, information sharing, and the definition of responsibilities in the care provided is relevant. Furthermore, this study suggests the need for patient involvement in safety practices, which could provide vital information for hospital management. It also suggests that developing countries, such as Brazil, should make progress in outlining patient involvement strategies for safer care that focus on the characteristics and needs of those receiving care.

## Conflicts of Interest

The authors declare no conflicts of interest.

## Data Availability

The data that support the findings of this study are available on request from the corresponding author. The data are not publicly available due to privacy or ethical restrictions.
